# Evaluation of the Novel RITA MTBC Assay for Tuberculosis Detection: A Pilot Comparison with GeneXpert and BD MAX™

**DOI:** 10.3390/pathogens15010021

**Published:** 2025-12-23

**Authors:** Tomasz Bogiel, Małgorzata Zimna, Renata Żebracka, Katarzyna Dziwnik, Monika Montowska, Dorota Krawiecka, Dawid Nidzworski, Marta Skwarecka, Kasjan Szemiako, Sabina Nidzworska, Marcin Woźniak, Kamil Drożdż, Agnieszka Krawczyk

**Affiliations:** 1 Department of Propaedeutics of Medicine and Infection Prevention Ludwik Rydygier Collegium Medicum in Bydgoszcz, Nicolaus Copernicus University in Toruń, 9 Maria Skłodowska-Curie Street, 85-094 Bydgoszcz, Poland; 2 Clinical Microbiology Laboratory, Dr. Antoni Jurasz University Hospital No. 1 in Bydgoszcz, 9 Maria Skłodowska-Curie Street, 85-094 Bydgoszcz, Poland; 3 Department of Microbiological Diagnostics, Kujawsko-Pomorskie Pulmonology Centre in Bydgoszcz, 85-326 Bydgoszcz, Poland; m.zimna@kpcp.pl (M.Z.); r.zebracka@kpcp.pl (R.Ż.); k.dziwnik@kpcp.pl (K.D.); m.montowska@kpcp.pl (M.M.); dorota.krawiecka@kpcp.pl (D.K.); 4 Institute of Biotechnology and Molecular Medicine, 25 Kampinoska Street, 80-180 Gdansk, Poland; d.nidzworski@ibmm.pl (D.N.); spibida.marta@gmail.com (M.S.); k.szemiako@ibmm.pl (K.S.); s.zoledowska@ibmm.pl (S.N.); 5 Department of Forensic Medicine, Ludwik Rydygier Collegium Medicum in Bydgoszcz, Nicolaus Copernicus University, 85-094 Bydgoszcz, Poland; marcinw@cm.umk.pl; 6 Department of Molecular Medical Microbiology, Jagiellonian University Medical College in Cracow, 31-121 Krakow, Poland; kamil.drozdz@uj.edu.pl

**Keywords:** *Mycobacterium tuberculosis*, molecular diagnostics, nucleic acid amplification tests, tuberculosis, rapid diagnostics, GeneXpert MTB/RIF, BD MAX™ MDR-TB

## Abstract

Tuberculosis is still one of the leading infectious causes of morbidity and mortality worldwide. Rapid diagnosis is essential for effective treatment and control of tuberculosis transmission. In recent years, nucleic acid amplification tests (NAATs), such as GeneXpert MTB/RIF, BD MAX™, Xpert MTB/RIF-Ultra, have significantly improved tuberculosis diagnostics. However, they mainly require expensive and advanced equipment. The aim of our study was to assess the usefulness of the novel RITA MTBC assay in this diagnostic context. A total of 61 clinical specimens were tested using the RITA MTBC assay in comparison with established molecular diagnostic platforms (GeneXpert and BD MAX™), used as molecular reference assays. Culture and microscopy were performed as part of initial clinical assessment, but the comparative analysis focused on molecular assays to provide a relevant evaluation of diagnostic performance. Among 31 samples previously identified as positive for *M. tuberculosis* DNA, the assay correctly detected 30 (LOT HPA01/20230601) and 29 (LOT HPA01/20230602). Of 30 negative samples, 28 and 30 were confirmed negative for the respective lots. These results correspond to an average sensitivity of 95% and an average specificity of 97%. The kit demonstrated diagnostic performance that meets requirements for molecular testing in tuberculosis, with sensitivity and specificity comparable to established platforms, although further validation on larger sample sets is necessary. Nevertheless, its excellent specificity, rapid turnaround time, and operational simplicity, make it especially well-suited for decentralized or resource-limited settings. These findings underscore the potential of RITA MTBC as a valuable diagnostic tool in both routine clinical settings and in populations with limited access to healthcare.

## 1. Introduction

**Figure 1:**
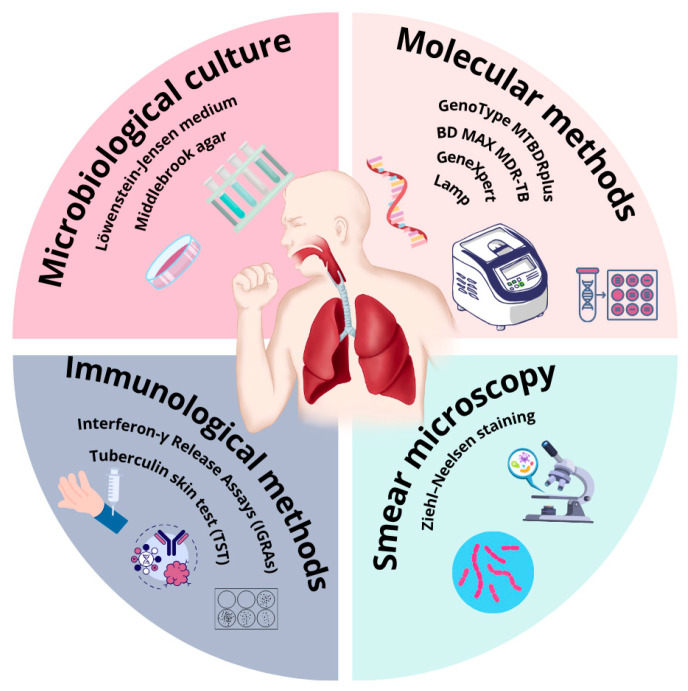
Overview of diagnostic methods used for the detection of tuberculosis.

Tuberculosis (TB) remains one of the leading infectious causes of morbidity and mortality worldwide, with over 10 million new cases and 1.3 million deaths annually [1]. Diagnosing and managing cases such as early-stage tuberculosis, extrapulmonary TB, co-infection with HIV, pediatric tuberculosis, and multidrug-resistant strains remains especially challenging. Rapid and accurate diagnosis is essential for effective treatment and control of TB transmission. Several diagnostic approaches are currently available for the detection of *Mycobacterium tuberculosis* (MTB), ranging from traditional methods to modern molecular assays (Figure 1). Traditional methods include microscopic evaluation of clinical samples (mostly sputum smears) and culture-based techniques, which, although highly informative, can be time-consuming. In contrast, molecular assays allow for a rapid and sensitive detection of MTB DNA.

In recent years, nucleic acid amplification tests (NAATs), such as GeneXpert MTB/RIF, BD MAX™ Multi Drug Resistant-Tuberculosis assay, Xpert^®^ MTB/RIF Ultra, and line probe assays (LPA), have significantly improved TB diagnostics by enabling detection of both *Mycobacterium tuberculosis* complex (MTBC) and drug resistance within 1–2 days of sample collection [2,3,4]. These assays are now widely recommended by the World Health Organization (WHO) and incorporated into routine TB diagnostic algorithms by national TB programs globally [1,5]. Their widespread adoption is largely due to several key advantages: they offer high analytical sensitivity and specificity, enable simultaneous detection of resistance to rifampicin and isoniazid, and provide results significantly faster than traditional culture-based methods. In particular, tests like Xpert MTB/RIF and Xpert Ultra have demonstrated utility in both pulmonary and extrapulmonary TB, and are effective even in cases of negative smear results, which are often missed by conventional microscopy. Furthermore, their automated and standardized nature reduces the risk of cross-contamination and operator error, enhancing reliability in routine diagnostic workflows [2,6]. Despite these advances, delays in diagnosis and initiation of appropriate therapy still occur, especially in resource-limited settings [7]. Moreover, the high cost and infrastructure requirements of existing NAATs limit their universal accessibility. These platforms often require stable electricity, controlled laboratory conditions, and trained personnel, which may not be readily available in decentralized or low-resource environments. Additionally, implementation of current NAATs at scale often encounters operational challenges, such as supply chain disruptions due to transport delays or extreme environmental conditions; the need to maintain a cold chain for reagents; and delays caused by centralized laboratory workflows [7]. These challenges are especially acute during public health emergencies or in remote regions, where logistical barriers significantly delay timely diagnosis. In such contexts, decentralized, user-friendly, and robust diagnostic platforms can help close the diagnostic gap and limit TB transmission. Therefore, there is an urgent need for innovative, rapid, affordable, and scalable diagnostics that enable timely detection and lead to better patient outcomes—particularly in environments where conventional molecular testing is difficult to implement [8,9].

The RITA by GeneMe (NxBiotech, Gdansk, Poland) is an innovative real-time PCR-based diagnostic test designed to overcome some of these limitations. The use of a novel, patented Taq polymerase combined with specially designed primers and probes has enabled the development of a highly specific and sensitive assay. Primers and probes are 100% compatible with *M. tuberculosis* genomic DNA sequence, specifically the insertion sequence IS6110 as referenced in the NCBI database, along with the human RNase P gene serving as an internal control. Although the assay requires prior DNA extraction, the input material does not need to be highly purified—a basic and rapid extraction procedure is sufficient. The preparation and run of the RITA MTBC test itself take approximately 40 min. The assay utilizes frozen reagents packaged in 8-well strips. These reagents are stable when stored frozen and, once thawed, remain usable for up to 6 h at 4–8 °C without significant loss of performance. This formulation combines the benefits of enhanced reagent stability with the flexibility required for efficient sample processing, allowing the test to maintain high sensitivity and specificity in various laboratory settings. Most importantly, compatibility with smaller, mobile PCR devices enables flexible deployment in decentralized or resource-limited settings, directly at the point of care, while its substantially lower cost compared with other molecular methods further enhances its suitability for widespread use. These features suggest that RITA could complement existing diagnostic tools by providing a rapid, cost-effective, and scalable alternative. Its flexible design and minimal infrastructure requirements (i.e., simple DNA extraction and a compact benchtop qPCR device) position it as a promising candidate for future point-of-care multiplex diagnostics.

The RITA MTBC assay is performed as an open molecular workflow and therefore requires standard laboratory biosafety precautions. According to the manufacturer’s instructions for use (in Supplementary Material), appropriate personal protective equipment and careful workspace organization are recommended to minimize contamination and staff exposure. Unlike fully closed cartridge-based systems such as GeneXpert, which minimize operator contact with clinical material after sample loading, the RITA workflow involves manual pipetting and handling of extracted DNA, representing a potential biosafety limitation. However, it should be emphasized that the RITA MTBC assay is performed on previously extracted DNA and does not involve manipulation of raw clinical material during PCR setup, resulting in a low biological risk at this stage. The open nature of the workflow requires rigorous practices to control potential contamination, rather than posing a significant biosafety concern. Operationally, the RITA MTBC assay offers flexibility and low infrastructure requirements, as it can be implemented using basic DNA extraction methods and a compact real-time PCR thermocycler. These features support its intended use as a complementary molecular diagnostic tool, particularly in decentralized or resource-limited settings where fully automated cartridge-based platforms may be unavailable.

From a public health perspective, shortening the diagnostic delay is key to controlling tuberculosis, particularly in high-burden regions. Innovative diagnostic tools, that provide reliable results in a short time and can be adapted to various conditions have the potential to accelerate progress toward global TB elimination goals in line with the World Health Organization’s End TB Strategy. In this context, evaluation of the effectiveness of RITA is essential to determine its viability as an alternative or complementary diagnostic method.

In light of the above, our study aimed to evaluate the effectiveness of the RITA MTBC test by GeneMe (NxBiotech, Gdańsk, Poland) for the identification of *M. tuberculosis* DNA in clinical samples. The primary objective of the experiment was to assess the rates of false positive and false negative results in comparison to the standard molecular tests BD MAX™ Multi Drug Resistant Tuberculosis and Gene Xpert^®^ MTB/RIF, which were used to calculate the sensitivity and specificity of the RITA MTBC test.

## 2. Materials and Methods

A total of 61 clinical specimens were collected between October 2022 and January 2023 from patients hospitalized in Kuyavian-Pomeranian Pulmonology Center in Bydgoszcz, Poland.

All clinical specimens were subjected to routine diagnostic procedures, including microscopy and culture, as part of the initial clinical assessment (Table S1). Based on these conventional methods, samples were preliminarily classified as *M. tuberculosis*-positive if either microscopy or culture gave a positive result, and as tuberculosis-negative if both methods gave a negative result. In most cases, true positive samples were confirmed by all available methods—culture, microscopy, and established molecular assays BD MAX™ Multi-Drug Resistant Tuberculosis (Becton Dickinson, Franklin Lakes, NJ, USA) and Gene Xpert^®^ MTB/RIF (Cepheid, Sunnyvale, CA, USA) while all the negative samples gave negative results in molecular testing.

To evaluate the analytical performance of the RITA MTBC assay (NxBiotech, Gdańsk, Poland), its results were compared with those obtained using well-established tuberculosis diagnostic platforms, such as BD MAX™ and GeneXpert. RITA MTBC was not directly compared with culture or microscopy results, as such a comparison would substantially underestimate its diagnostic performance and could be misleading. Molecular assays are known to be more sensitive than conventional methods, so comparing RITA solely to culture or microscopy could falsely suggest “over-detection” whereas this difference actually reflects the higher analytical sensitivity of molecular diagnostics. This approach allows for a meaningful assessment of RITA’s diagnostic accuracy relative to widely used, WHO-recommended nucleic acid amplification tests, which currently represent the standard of care for rapid tuberculosis diagnosis.

All samples were stored at −80 °C until molecular testing was conducted. The specimens included a variety of sample types: bronchoalveolar lavage (33 samples), sputum (21 samples), pleural fluid (5 samples), tissue biopsy (1 sample), and gastric fluid (1 sample). In total, 30 samples were classified as negative and 31 as positive, according to the manufacturer’s declared limit of detection of the kit, namely 80 CFU/mL. According to the comparison with BD MAX™ MDR-TB or Gene Xpert^®^ MTB/RIF the positive samples contained a pathogen concentration (at least 80 CFU/mL). Samples with a high level of PCR inhibitors—characterized by dense, cloudy appearance or visible blood contamination, were excluded from the analysis.

Prior to analysis, DNA was manually extracted using minicolumns. The extraction was performed with a commercially available kit compliant with In Vitro Diagnostic Regulation (IVDR)—the GeneProof PathogenFree DNA Isolation Kit (GeneProof, Brno, Czech Republic), Ref. IDNA050. Isolated DNA from each sample was tested with the RITA MTBC assay using two different LOT numbers (LOT: HPA01/20230601 and LOT: HPA01/20230602), each in a single technical replicate.

All the steps were performed in the class II biosafety cabinets. Prior to assay preparation, all reagents, including the 2xMasterMix qPCR Probe, RITA-Oligos Set, and Nuclease-Free Water, were thawed at room temperature and maintained on ice to preserve stability. The 2xMasterMix qPCR Probe and RITA Oligos Set tubes were briefly vortexed and centrifuged before use. A reaction mixture was prepared by combining 10 μL of 2xMasterMix qPCR Probe, 5 μL of RITA-Oligos Set, and 1 μL of Nuclease-Free Water per reaction. Sixteen microliters of this mixture were then dispensed into each PCR well. For negative control wells, 4 μL of Nuclease-Free Water was added to the reaction mixture intended for a negative control, whereas positive control wells were supplemented with 4 μL of the RITA Positive Control. All transfers were performed using sterile filtered tips, and the wells were immediately sealed after addition of the respective controls. Extracted DNA samples were mixed by brief vortexing and 4 μL of each sample was transferred to the corresponding reaction wells, and the plate was immediately sealed. Prepared plates were briefly centrifuged in a mini-centrifuge to ensure all liquid collected at the bottom of the wells and to remove any air bubbles. The plates were then placed in CFX96 Opus Dx Real-Time Detection System (Bio-Rad, Inc., Hercules, CA, USA) in the correct orientation according to the sample layout. The reaction was carried out according to the thermal cycling program presented in Table 1, with fluorescence detection conducted in the FAM channel (498/522 nm) and HEX channel (538/554 nm). If preparation exceeded 30 min, the strips or plates were stored at 4–8 °C for no longer than 3 h before amplification.

Run validity was confirmed by the expected signals in the control wells: negative controls showed no signal (Cq undetermined) and positive controls exhibited a Cq < 30 in both FAM and HEX channels (Table 2). Only runs meeting these criteria were considered valid, and sample results were interpreted according to the manufacturer’s interpretation guidelines, as summarized in Table 3. All the procedures were performed following the kit instructions, maintaining strict adherence to contamination prevention and temperature control throughout the assay.

### Statistical Analysis

Statistical analysis was performed using IBM SPSS Statistics software, version 29.0.2 (IBM Corp., Armonk, NY, USA). The consistency of the results obtained for the two reagent series (HPA01/20230601 and HPA01/20230602) was assessed using paired data. Differences between the series were analyzed using the exact McNemar test based on the binomial distribution, which was justified by the small number of discordant pairs. This test was used to evaluate the hypothesis of symmetry in discrepancies between the results obtained for the two series. Additionally, the degree of agreement between the reagent series was assessed using Cohen’s kappa coefficient, which accounts for observed agreement adjusted for agreement occurring by chance. Kappa coefficient values were interpreted according to generally accepted criteria. Furthermore, basic diagnostic performance parameters were calculated, including sensitivity, specificity, positive and negative predictive values (PPV and NPV), and overall accuracy, along with their 95% confidence intervals. Results with a *p*-value < 0.05 were considered statistically significant.

## 3. Results

A total of 61 clinical specimens were analyzed using the RITA MTBC assay in accordance with the instructions. Samples were interpreted as positive for *M. tuberculosis* DNA when FAM Cq < 40 and HEX Cq < 40 or undetermined; negative results were defined as FAM Cq undetermined and HEX Cq < 40. Reactions were considered undiagnostic if both FAM and HEX signals were undetermined (Table 3).

Among the 31 samples previously identified as positive for *M. tuberculosis* DNA using molecular methods (BD MAX™ MDR-TB or Gene Xpert^®^ MTB/RIF), the RITA MTBC assay with LOT number HPA01/20230601 gave 30 positive and one negative result. When the same set of positive samples was tested with another batch of reagents (LOT no. HPA01/20230602), 29 samples appeared positive, while two results were negative.

Out of the 30 samples previously determined to be negative, 28 were confirmed negative using LOT HPA01/20230601, whereas all 30 tested negative with LOT HPA01/20230602. Notably, two samples gave positive results when tested with LOT HPA01/20230601, despite being part of the negative sample group.

The obtained results enabled the evaluation of the diagnostic performance of the RITA MTBC test. High sensitivity and specificity were observed for both production lots, with comparable performance across lots. The detailed statistical parameters are presented in Table 4.

Agreement between HPA01/20230601 and HPA01/20230602 was observed in 95.1% (29 + 29/61) of cases, with only 4.9% (0 + 3/61) discordant results. The exact McNemar test showed no statistically significant differences between the two reagent lots (*p* = 0.250). In addition, agreement analysis demonstrated an almost perfect concordance, as indicated by Cohen’s kappa (κ = 0.902, *p* < 0.001).

**Figure 2:**
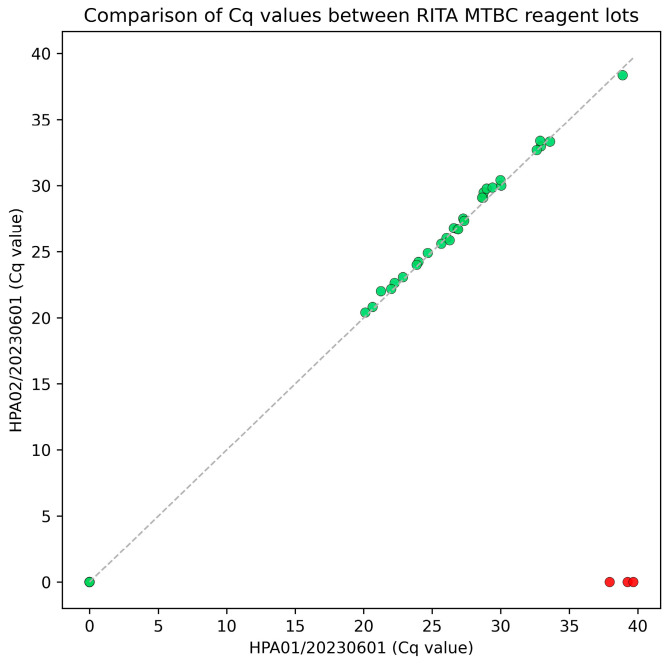
Scatter plot comparing Cq values obtained using two different production lots of the RITA MTBC assay. Green dots indicate concordant results between lots, while red dots represent discordant data points.

A scatter plot was generated to compare Cq values from the two production lots, demonstrating high agreement with only three outlying data points (Figure 2).

Although high diagnostic performance and excellent agreement between reagent lots were observed, the limited sample size restricted the statistical power to formally demonstrate sensitivity and specificity values exceeding predefined thresholds and to exclude small lot-to-lot differences

**Figure 3:**
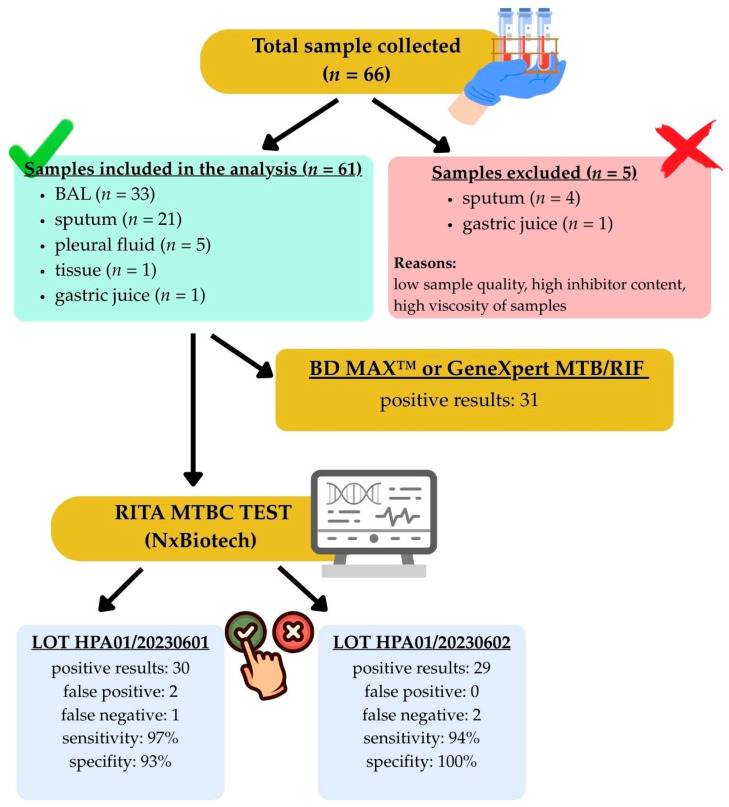
Flow chart of clinical sample processing illustrating the final positive and negative classifications obtained with the RITA MTBC assay (NxBiotech) for two independent lots, in comparison with BD MAX™ and GeneXpert^®^ reference assays.

A flow diagram illustrating sample collection, exclusion criteria, final inclusion in the analysis, and the ultimately obtained test results is presented in Figure 3.

## 4. Discussion

Although culture and microscopy remain classical reference methods for tuberculosis diagnosis, they were not described as the primary reference assays in this study. The analysis focused on molecular performance, and therefore RITA MTBC results were compared to established NAAT platforms (BD MAX™ and Gene Xpert) serving as a molecular reference standard. This approach reflects current molecular diagnostic algorithms and clinical practice, as molecular assays are more sensitive than conventional methods. Comparing RITA solely with culture or microscopy could misleadingly suggest “over-detection” by RITA, whereas such differences actually reflect the higher analytical sensitivity of molecular diagnostics. If the objective of this study had been to compare the performance of molecular assays with conventional methods, such as culture and microscopy, these classical methods would have been included in the analysis. However, the focus of the present work was specifically to evaluate the diagnostic performance of RITA MTBC in the context of molecular testing, and therefore the assessment was performed in comparison to established NAAT reference assays only.

With an average sensitivity of 95% and specificity of 97%, the RITA assay demonstrates diagnostic accuracy comparable to that of established molecular platforms such as GeneXpert^®^ and BD MAX™ [2,6,7,10,11]. Importantly, these results suggest that RITA may provide a reliable alternative for TB detection in clinical settings, achieving sensitivity and specificity comparable to widely used commercial assays. The rapid workflow, low cost, and minimal infrastructure requirements (i.e., simple DNA extraction and a compact benchtop qPCR device) make this assay particularly suitable for decentralized or resource-limited environments. However, it should be noted that the assay is not intended for drug-resistance testing.

In the context of reported data for established molecular platforms, we summarize below the sensitivity and specificity values for Gene Xpert MTB/RIF, Xpert MTB/RIF Ultra, and BD MAX, previously described in the literature, to serve as a reference point for the interpretation of RITA MTBC test results. For instance, Dorman et al., in their multicenter analysis, showed that the mean sensitivity for the Xpert test was 83%, while for the Xpert^®^ MTB/RIF Ultra it was 88%. The specificity was 98% and 96%, respectively [12].

In another independent study by Hueda-Zavaleta et al., involving 1023 clinical samples, the performance of both Xpert^®^ MTB/RIF and Xpert^®^ MTB/RIF Ultra was evaluated across multiple specimen types. The assays demonstrated a combined sensitivity and specificity of 97% and 93% in pulmonary samples, 100% and 98.3% in cerebrospinal fluid, 66.7% and 96.8% in pleural fluid, and 100% and 94.3% in urine, respectively. For the detection of pulmonary tuberculosis specifically, the Xpert^®^ MTB/RIF assay achieved a sensitivity of 97.1% and specificity of 95.6%, while the Xpert^®^ MTB/RIF Ultra showed a sensitivity of 97% and specificity of 89.5% [6]. By comparison, the RITA assay’s performance across respiratory samples in our study remained consistently high, reinforcing its potential for reliable TB detection regardless of specimen type. These results highlight some variability in specificity depending on sample type, emphasizing that appropriate sample selection and proper collection techniques are crucial factors to consider in clinical decision-making.

Shah et al. [13], in a large-scale cohort of 1053 participants with presumptive TB, reported that the BD MAX™ assay showed 93% sensitivity and 97% specificity in culture-negative sputum, 100% in smear-positive, and 81% in smear-negative samples. Among participants tested with both BD MAX™ and Xpert^®^ assays, sensitivities were comparable, with BD MAX™ kit achieved 91% and Xpert assay—90% on processed sputum samples. Overall, our findings demonstrate that the RITA MTBC assay provides results comparable to well-established, standardized molecular tests/platforms such as Xpert^®^ MTB/RIF Ultra and BD MAX™ MDR-TB, while significantly reducing turnaround time and simplifying sample processing. However, unlike these platforms, RITA does not detect drug resistance, and is therefore intended solely for the rapid identification of the presence of *M. tuberculosis* DNA.

When comparing RITA MTBC to other, less widely adopted molecular diagnostic tools reported in the literature, such as Truenat MTB, Abbott Real Time, TB Lamp, AdvanSure TB/NTM real-time PCR assay [14,15,16,17,18], the diagnostic performance of our test appears favorable, particularly in terms of turnaround time and operational simplicity, without compromising its high sensitivity and specificity. The RITA MTBC test offers a simple, low-cost, and portable molecular diagnostic solution that can be performed with minimal laboratory infrastructure, using compact thermocyclers and standard DNA extraction methods. Importantly, the overall cost of performing the RITA assay is expected to be substantially lower than that of commercial platforms. Although precise calculations will only be possible after the commercialization phase, the reduced assay complexity and low-cost reagent formulation indicate that such a reduction is feasible. Future development of the assay will also include the integration of resistance-associated mutation detection, further extending its diagnostic utility. In preliminary experiments, lyophilized reagents stored at room temperature were also evaluated. However, the frozen formulation demonstrated superior performance in terms of sensitivity and reproducibility. Notably, once thawed, the reagents remain stable for up to 6 h at 4–8 °C, which is sufficient for typical laboratory workflows and does not limit the assay’s flexibility. This approach combines the benefits of enhanced reagent stability with practical handling requirements, ensuring reliable results in routine diagnostic settings. For comparison, commonly used molecular TB platforms exhibit different storage and stability profiles: GeneXpert cartridges and BD MAX reagents are stored at 2–28 °C; Truenat TB chips can be stored for up to 6 months at temperatures below 40 °C; and Abbott RealTime reagents require storage at –15 to –25 °C.

To better highlight the advantages of the RITA MTBC assay, it is useful to compare it with other molecular diagnostic tools designed for similar applications. For instance, Ssengooba et al. assessed the performance of the Truenat MTB assay [14], a chip-based PCR platform delivering results in less than an hour, demonstrating 81% sensitivity and 94% specificity compared to Xpert^®^ MTB/RIF Ultra. In turn, a meta-analysis of nine studies involving 3640 patients showed that a different test—Abbott Real Time [15], achieved higher sensitivity (97%) and specificity (96%) but requires longer processing times and complex infrastructure. Although the Abbott assay has the added benefit of detecting antibiotic resistance, the longer turnaround time and the need for complex equipment requiring a large infrastructure make the RITA MTBC a more suitable option for rapid, decentralized testing with comparable accuracy.

Another, less popular diagnostic option is TB-LAMP, an isothermal amplification test for sputum that does not require sophisticated equipment and the time to obtain the result takes only 2 h. In a multicenter study TB-LAMP showed moderate overall sensitivity (75.6%), with high sensitivity in smear-positive samples (97.9%) but limited performance in smear-negative cases (46.6%), while maintaining high specificity (98.7%) [16]. In comparison, the RITA MTBC assay in our study achieved higher overall sensitivity (95%), particularly in smear-negative samples, with similarly high specificity and faster time to result.

It is important to note that samples with high viscosity, dense appearance, or visible blood contamination were excluded from the present study, in line with the manufacturer’s instructions for the RITA MTBC assay. Specifically, 5 out of 66 collected specimens (7.6%) were excluded for these reasons (Figure 3; Supplementary Table S2). Especially dense or highly viscous samples can contain PCR polymerase inhibitors, which may affect assay performance. In routine practice, such samples would require pre-treatment, such as homogenization, liquefaction, or dilution, before testing to ensure accurate results. Although a few tests were conducted on such dense or blood-contaminated specimens, the results were sometimes unreliable, highlighting that the exclusion of these challenging specimens may lead to a slight overestimation of assay performance in our study. Future development of the RITA MTBC assay will take these limitations into account, and dedicated analyses on technically demanding specimens are planned to optimize assay performance under real-world settings. This supports the need to recognize this limitation of the assay. Future studies should specifically evaluate the performance of RITA MTBC on dense or complex clinical specimens to establish standardized handling procedures and verify assay reliability in these challenging sample types.

It should also be noted that the present study included a relatively small number of clinical specimens (*n* = 61). While this sample size is limited, it is typical for initial evaluations of new diagnostic assays aimed at assessing feasibility, preliminary performance, and operational aspects. Despite the limited number, the study provides meaningful insights into the sensitivity and specificity of the RITA MTBC assay and highlights its potential for rapid, decentralized TB diagnostics. It is important to emphasize that access to original clinical samples used in TB diagnosis is very limited and highly restricted. Unlike artificially prepared or intentionally enriched materials, obtaining true positive samples for tuberculosis is difficult due to stringent biosafety regulations and the need to avoid contamination that could compromise diagnostic integrity. International public health agencies, including WHO and ECDC, exercise rigorous oversight and often centralize TB diagnostic procedures to ensure biosafety, standardization, and prevention of laboratory contamination. As a result, the availability of authentic, diagnostically relevant TB samples for research and validation studies is severely limited. In this context, the number of samples included in our study is significant, especially considering that we were able to perform repeat analyses on two reagent lots, which allowed for a more reliable assessment of assay reproducibility than is typically possible with early-stage assessments. Nevertheless, we recognize that larger, multi-center studies will be required to confirm these findings and improve the generalizability and robustness of the results across diverse patient populations and sample types. Despite the overall high concordance between RITA MTBC and the reference molecular assays, a small number of inconsistent results were observed and are summarized in Supplementary Table S1. Considering the reasons for the slightly lower sensitivity observed with LOT HPA01/20230602 (94%) compared to LOT HPA01/20230601 (97%), this difference may be due to minor reagent lot-to-lot variation, such as small differences in primer or probe performance, enzyme activity (e.g., Taq polymerase), or reagent stability between production batches, even when using identical DNA templates [19]. Additionally, even small deviations in reagent lyophilization, drying temperature, or storage humidity during production could have influenced enzyme conformation or primer-probe efficiency, resulting in slightly reduced amplification performance in one of the lots [20,21,22,23,24].

However, it is also possible that the occurrence of false-negative results in LOT HPA01/20230602 was influenced by the presence of PCR inhibitors in the clinical sample. Although both reagent lots were tested on the same DNA isolates, differences in reagent composition or tolerance to inhibitors may have affected amplification efficiency [24]. PCR inhibition is a well-documented challenge in tuberculosis diagnostics and may impact the performance of highly sensitive molecular tests, particularly depending on the type of clinical specimen being tested. Such issues may contribute to occasional discordant results [22,24]. In our studies, we observed that some sample types, such as sputum, gave fully consistent results across all tests, while other sample types showed occasional minor discrepancies.

One discordant result was observed in a bronchoalveolar lavage (BAL) specimen that tested negative by culture, microscopy, BD MAX™, and one RITA MTBC reagent lot (HPA01/20230602), whereas the second RITA lot (HPA01/20230601) gave a positive result. All other diagnostic methods for this specimen were consistently negative, suggesting a single false-positive result, possibly caused by lot-specific reagent variation or non-specific signal generation [20,23,25].

A similar false-positive result with the same RITA reagent lot (HPA01/20230601) was observed in a gastric juice sample, for which all other methods (including culture, microscopy, BD MAX™, and RITA tested with the alternative lot) were negative. Gastric juice is a challenging specimen type due to variable acidity and inhibitory substances, which may affect assay performance [26]. The discordant result was observed only with one reagent lot, while the second lot gave a result consistent with all other diagnostic methods. Such lot-dependent variability, although infrequent, has been reported for highly sensitive molecular assays and may become apparent particularly in challenging specimens. Minor discrepancies between runs or reagent batches are well recognized in molecular diagnostics, and testing in duplicate is recommended.

A similar pattern was observed in a BAL sample, where both RITA reagent lots, as well as culture and microscopy, gave negative results, whereas Gene Xpert showed a positive result. This finding further supports our previous observations that minor discrepancies between assays (or reagent lots) can occur, particularly in samples where bacterial load is low or the sample contains interfering substances [19,20,27].

Another discordant result was identified in a pleural fluid specimen, which tested negative by microscopy, showed only weak positivity in culture (single “+”), and was positive by BD MAX™. This pattern likely reflects very low bacterial load typically present in pleural fluid, which can undermine sensitivity of any diagnostic assay. These findings underscore the importance of interpreting molecular test results in combination with complementary diagnostic methods, particularly for specimen types that are known to contain few microorganisms or that are technically difficult to detect [27].

Overall, in the present research, discordant results were rare and were mostly observed in specimen types that are inherently challenging for TB diagnosis, such as BAL, gastric juice, and pleural fluid. Despite these occasional discrepancies, the RITA MTBC assay demonstrated consistently high sensitivity and specificity across the majority of samples, highlighting its robust performance in detecting *M. tuberculosis* complex in diverse clinical specimens.

Compared to traditional culture methods, which can take weeks to obtain results, rapid molecular assays offer a significant advantage in reducing the diagnostic delay—a key objective of the WHO End TB Strategy [1,8]. The RITA MTBC assay, which provides results in approximately 40 min and requires only simple sample preparation rather than full DNA purification, is well-suited not only for rapid tuberculosis diagnosis, but also for use in decentralized or resource-constrained environments. These features align with WHO’s target product profile for TB diagnostics aimed at use at the point of care [1]. Importantly, the RITA assay requires minimal equipment requirements, and is compatible with portable PCR devices making it particularly adaptable to field conditions, which is a known limitation of many current molecular diagnostic platforms [9,10].

While the current study is limited by a relatively small sample size, the results are promising and suggest that RITA MTBC could serve as a complementary tool within national TB diagnostic algorithms, especially in settings with limited access to centralized laboratories. Further large-scale, multi-center evaluations in diverse epidemiological contexts will be necessary to confirm the generalizability and operational feasibility of this assay, as well as further approaches to implementing another aspect of TB diagnostics—the detection of antimicrobial resistance. Additionally, future studies should include multiple technical replicates for each reagent lot to provide a more precise assessment of intra-lot variability and assay reproducibility. Moreover, although our study included mixed sample types (BAL, sputum, and pleural fluid), the number of specimens for each type was limited, which precluded formal stratified performance analyses. Nevertheless, testing across different specimen types provided valuable insights into how the RITA MTBC assay performs on a variety of clinical materials, and future studies will include a larger number of samples from different biological materials.

## 5. Conclusions

The RITA MTBC assay demonstrated promising diagnostic performance broadly consistent with WHO-recommended standards for molecular testing in tuberculosis, with sensitivity and specificity comparable to established platforms and assays such as Xpert^®^ MTB/RIF and BD MAX™ MDR-TB, although larger-scale studies are needed to confirm these findings. Nevertheless, its excellent specificity, low cost, rapid turnaround time (approximately 40 min), and operational simplicity, make it especially well-suited for decentralized or resource-limited settings. Although slight variability between reagent lots was observed, likely due to minor manufacturing differences or the presence of PCR inhibitors, the overall stability and reliability of the RITA assay were maintained. These findings underscore the potential of RITA MTBC as a valuable diagnostic tool in both routine clinical settings and in populations with limited access to healthcare.

## 6. Patents

Instructions for Use (IFU) of the commercially established kit are provided as the Supplementary Material.

## Figures and Tables

**Table 1:** Thermal cycling program for the RITA Detection Kit assay.

Temperature	Time	Cycles
95 °C	30 s	1
95 °C	5 s	40
60 °C	15 s	40

**Table 2:** Interpretation of the controls.

Type	Negative Control	Positive Control
FAM	No signal (Cq undetermined)	Cq < 30
HEX	No signal (Cq undetermined)	Cq < 30
Interpretation	Valid	Valid

**Table 3:** Qualitative interpretation of the results for samples tested with the RITA MTBC assay.

Detection Channel	Cq	Interpretation
FAM HEX	Cq undetermined Cq < 40	NEGATIVE—absence of *Mycobacterium tuberculosis* DNA in the tested sample
FAM HEX	Cq < 40 Cq < 40 or Cq undetermined	POSITIVE—presence of *Mycobacterium tuberculosis* DNA in the tested sample
FAM HEX	Cq undetermined Cq undetermined	UNDIAGNOSTIC (low sample quality)

**Table 4:** Diagnostic performance of the RITA MTBC assay evaluated for two production lots (HPA01/20230601 and HPA01/20230602).

Parameter	RITA MTBC HPA01/20230601	RITA MTBC HPA01/20230602	Combined Results (Both LOTs)
N	61	61	61
TP	30	29	30
TN	28	30	28
FP	2	0	2
FN	1	2	1
Sensitivity (95% CI)	97% (83–99)	94% (79–98)	97% (84–100)
Specificity (95% CI)	93% (79–98)	100% (89–100)	93% (79–98)
PPV (95% CI)	0.94 (0.80–0.98)	1.00 (0.88–1.00)	0.94 (0.80–0.98)
NPV (95% CI)	0.97 (0.83–0.99)	0.94 (0.80–0.98)	0.97 (0.83–0.99)
Accuracy (95% CI)	95% (87–98)	97% (89–100)	95% (87–98)
LR+	14.52	–	14.52
LR−	0.03	0.06	0.03
Youden’s J	0.9	0.94	0.9

## Data Availability

The data presented in this study are available upon request from the corresponding author.

## Supplementary Materials

The following supporting information can be downloaded at: https://www.mdpi.com/article/10.3390/pathogens15010021/s1, RITA RITA MTBC test Instruction for use Kit and Supplementary Material containing Table S1: Comparison of results obtained for clinical samples using culture, microscopy, and molecular methods and the RITA MTBC test (inconsistent results highlighted in red); and Table S2: Clinical specimens excluded from the analysis and reasons for exclusion.

## Abbreviations

The following abbreviations are used in this manuscript:

BD MAX™	BD MAX™ device (Becton Dickinson)
BD MAX™ MDR-TB	BD MAX™ Multi Drug Resistant Tuberculosis assay
GeneXpert^®^	GeneXpert^®^ device (Cepheid)
Xpert^®^ MTB/RIF Ultra	Xpert^®^ MTB/RIF Ultra assay (Cepheid)
NAATs	nucleic acid amplification tests
TB	tuberculosis
