# Supplementary material for: Evaluation of the Novel RITA MTBC Assay for Tuberculosis Detection: A Pilot Comparison with GeneXpert and BD MAX™
Source: Pathogens. 2025 Dec 23;15(1):21. doi: 10.3390/pathogens15010021 (PMC12844691; doi:10.3390/pathogens15010021)
Supplement: Supplementary file 1 [file pathogens-15-00021-s001.zip › pathogens-4046286-supplementary.pdf]

pathogens

MDPI

Article

Evaluation of the novel RITA MTBC assay for tuberculosis detection: a pilot comparison with GeneXpert and BD MAX™

Tomasz Bogiel [1,2,*], Małgorzata Zimna [3], Renata Żebracka [3], Katarzyna Dziwnik [3], Monika Montowska [3], Dorota Krawiecka [3], Dawid Nidzworski [4], Marta Skwarecka [4], Kasjan Szemiako [4], Sabina Nidzworska [4], Marcin Woźniak[5], Kamil Drożdż[6], and Agnieszka Krawczyk [6,*]

Table S1. Comparison of results obtained for clinical samples using culture, microscopy and molecular methods and the RITA MTBC test; inconsistent results highlighted in red.

			Diagnostic reference methods				RITA MTBC assay	
No.	Sample name	Type of sample	AFB	Culture	Reference Molecular Method Used	Result of molecular assay [Ct]	Result for LOT HPA01/20230601 [Ct]	Result for LOT HPA01/20230602 [Ct]
1	U1	BAL	neg	neg	BD MAX	0.0	0.0	0.0
2	U2	BAL	neg	neg	BD MAX	0.0	0.0	0.0
3	U3	BAL	neg	neg	BD MAX	0.0	0.0	0.0
4	U4	BAL	neg	neg	BD MAX	0.0	0.0	0.0
5	U5	BAL	neg	neg	BD MAX	0.0	0.0	0.0
6	U6	BAL	neg	neg	BD MAX	0.0	0.0	0.0
7	U7	sputum	neg	neg	BD MAX	0.0	0.0	0.0
8	U8	BAL	neg	neg	BD MAX	0.0	0.0	0.0
9	U9	BAL	neg	neg	BD MAX	0.0	0.0	0.0
10	U10	BAL	neg	neg	BD MAX	0.0	0.0	0.0
11	U11	BAL	neg	neg	BD MAX	0.0	0.0	0.0
12	U12	BAL	neg	neg	BD MAX	0.0	0.0	0.0
13	U13	BAL	neg	neg	BD MAX	0.0	0.0	0.0
14	U14	BAL	neg	neg	BD MAX	0.0	37.94 **[FP]**	0.0
15	U15	BAL	neg	neg	BD MAX	0.0	0.0	0.0
16	U16	BAL	neg	neg	BD MAX	0.0	0.0	0.0
17	U17	BAL	neg	neg	BD MAX	0.0	0.0	0.0
18	U18	sputum	neg	neg	BD MAX	0.0	0.0	0.0
19	U19	BAL	neg	neg	BD MAX	0.0	0.0	0.0
20	U20	BAL	neg	neg	BD MAX	0.0	0.0	0.0
21	U21	sputum	neg	neg	BD MAX	0.0	0.0	0.0
22	U22	BAL	neg	neg	BD MAX	0.0	0.0	0.0
23	U23	pleural fluid	neg	neg	BD MAX	0.0	0.0	0.0
24	U24	BAL	neg	neg	BD MAX	0.0	0.0	0.0
25	U25	gastric juice	neg	neg	BD MAX	0.0	39.25 **[FP]**	0.0
26	U26	BAL	neg	neg	BD MAX	0.0	0.0	0.0
27	U27	BAL	neg	neg	BD MAX	0.0	0.0	0.0

 https://doi.org/10.3390/xxxxx

28	U28	pleural fluid	neg	neg	BD MAX	0.0	0.0	0.0
29	U29	pleural fluid	neg	neg	BD MAX	0.0	0.0	0.0
30	U30	tissue frag.	neg	neg	BD MAX	0.0	0.0	0.0
31	P1	sputum	+	+	GX	16.3	28.73	29.08
32	P3	BAL	+++	+	GX	16.0	24.00	24.22
33	P4	sputum	+++	+	GX	16.1	20.65	20.82
34	P6	BAL	++	+	GX	16.1	22.87	23.07
35	P7	sputum	++	+	GX	16.4	28.76	29.48
36	P8	sputum	neg	+	GX	18.6	32.94	32.96
37	P9	sputum	+++	+	GX	15.9	26.05	26.02
38	P10	BAL	neg	neg	GX	26.9	0.0 **[FN]**	0.0 **[FN]**
39	P11	BAL	+++	+	BD MAX	22.2	24.68	24.89
40	P12	sputum	+++	+	BD MAX	23.8	26.82	26.71
41	P13	BAL	+++	+	GX	16.3	25.67	25.59
42	P15	sputum	+++	+	BD MAX	21.9	26.58	26.78
43	P17	BAL	+	+	BD MAX	28.7	33.59	33.32
44	P18	sputum	+	+	BD MAX	28.6	38.89	38.35
45	P21	BAL	neg	+	BD MAX	30.3	32.64	32.68
46	P22	sputum	+++	+	BD MAX	24.8	28.98	29.77
47	P23	sputum	+++	+	BD MAX	20.9	20.12	20.40
48	P24	BAL	+	+	BD MAX	26.9	29.41	29.85
49	P25	BAL	+++	+	BD MAX	26.8	27.27	27.51
50	P26	sputum	+	+	GX	16.1	30.05	30.00
51	P27	sputum	++	+	GX	16.4	32.89	33.38
52	P28	sputum	+++	+	BD MAX	18.9	21.26	22.01
53	P30	BAL	+	+	BD MAX	29.7	29.99	30.41
54	P31	sputum	+++	+	BD MAX	23.6	26.90	26.69
55	P33	BAL	+++	+	GX	16.1	22.26	22.63
56	P34	sputum	+++	+	BD MAX	27.7	28.65	29.09
57	P35	sputum	+++	+	BD MAX	22.5	23.87	24.00
58	P36	pleural fluid	neg	+	BD MAX	33.4	39.67	0.0 **[FN]**
59	P37	sputum	+++	+	BD MAX	21.3	22.01	22.18
60	P38	sputum	++	+	BD MAX	29.1	27.34	27.34
61	P40	pleural fluid	+	neg	BD MAX	26.1	26.28	25.85

AFB – Acid-fast bacillus test; BAL – bronchoalveolar lavage samples; BD MAX –BD MAX Multi Drug Resistant Tuberculosis; GX - GeneXpert MTB/RIF; FN – false negative; FP – false positive; tissue frag. – tissue fragments

Table S2. Clinical specimens excluded from analysis and reasons for exclusion.

Sample name	Type of sample	Observations	Comment	Interpretation
P20	sputum	-	technical error, incorrect isolation process	not analyzed
P2	sputum, sample with a brown tint	None FAM signal for both LOT numbers of the RITA MTBC test	low sample quality, presence of inhibitors originating from blood	negative, excluded from analysis
P5	sputum, sample with a brown tint	FAM signal for both LOT numbers of the RITA MTBC test	low sample quality, presence of inhibitors originating from blood	positive, excluded from analysis
P14	sputum, sample with a brown tint	FAM signal for one LOT number of the RITA MTBC test	low sample quality, presence of inhibitors originating from blood	inconclusive, excluded from analysis
P29	gastric juice, dense sample with a brown tint	FAM signal for both LOT numbers of the RITA MTBC test	low sample quality, presence of inhibitors originating from blood	positive, excluded from analysis
